# Embodiment, tailoring, and trust are important for co‐construction of meaning in physiotherapy after stroke: A qualitative study

**DOI:** 10.1002/pri.1948

**Published:** 2022-03-20

**Authors:** Marianne Sivertsen, Hanne De Jaegher, Ellen Christin Arntzen, Karl Bjørnar Alstadhaug, Britt Normann

**Affiliations:** ^1^ Department of Health and Care Sciences UiT The Arctic University of Norway Tromsoe Norway; ^2^ Nordland Hospital Trust Bodoe Norway; ^3^ Department of Philosophy University of the Basque Country San Sebastián Spain; ^4^ School of Psychology University of Sussex Brighton UK; ^5^ Faculty of Nursing and Health Sciences Nord University Bodoe Norway; ^6^ Department of Clinical Medicine UiT The Arctic University of Norway Tromsoe Norway

**Keywords:** enactive theory, interaction, physiotherapy, qualitative research, stroke

## Abstract

**Background and Purpose:**

Physiotherapy, with an emphasis on high intensity, individually tailored, and person‐centered treatment, is an effective route for recovery after a stroke. No single approach, however, has been deemed paramount, and there is limited knowledge about the patient experience of assessment, goal‐setting, and treatment in physiotherapy. In this study, we seek to report patient experiences of I‐CoreDIST—a new physiotherapy intervention that targets recovery—and those of usual care. The purpose is to investigate how individuals with stroke experience the bodily and interactive course of physiotherapy during their recovery process.

**Methods:**

A qualitative study, nested within a randomized controlled trial, consisting of in‐depth interviews with 19 stroke survivors who received either I‐CoreDIST or usual care. Data were analyzed using systematic text condensation, and this analysis was informed by enactive theory.

**Results:**

Interaction with the physiotherapist, which was guided by perceived bodily changes, fluctuated between being, on the one hand, formal/explicit and, on the other, tacit/implicit. The experiences of participants in the intervention group and the usual care group differed predominantly with regards to the content of therapy sessions and the means of measuring progress; divergences in levels of satisfaction with the treatment were less pronounced. The perception of positive bodily changes, as well as the tailoring of difficulty and intensity, were common and essential features in generating meaning and motivation. An embodied approach seemed to facilitate sense‐making in therapy situations. In the interaction between the participants and their physiotherapists, trust and engagement were important but also multifaceted, involving both interpersonal skills and professional expertise.

**Conclusion:**

The embodied nature of physiotherapy practice is a source for sense‐making and meaning‐construction for patients after a stroke. Trust in the physiotherapist, along with emotional support, is considered essential. Experiencing progress and individualizing approaches are decisive motivators.

## 1 INTRODUCTION

Physiotherapy is effective for the recovery of function and mobility after a stroke (Pollock et al., 2014). High‐dose and high‐intensity training, together with selecting treatment components based on an individual assessment, are recommended as a foundation for implementing evidence‐based treatment (Pollock et al., 2014; Saunders et al., 2020). Several treatment approaches exist, but no single one has been elevated as superior to any other (Bernhardt et al., 2017; Pollock et al., 2014). Additionally, patient experiences of physiotherapy assessment after a stroke are insufficiently investigated (Pak et al., 2015). New interventions promoting recovery, as opposed to compensatory strategies, are called for (Frykberg & Vasa, 2015; Levin & Demers, 2020). I‐CoreDIST^1^ is a recent, individualized intervention aimed at recovery. For people with multiple sclerosis, it has proved effective (Arntzen, Straume, et al., 2019) and meaningful in group settings (Arntzen, Oberg, et al., 2019). It is also feasible in individual post‐stroke rehabilitation (Normann et al., 2019). How this intervention is perceived by individuals with sub‐acute stroke has not yet been investigated. Moreover, there is generally limited knowledge about patient perspectives on the content and impact of usual care physiotherapy after a stroke. In the development of new interventions, it is vital to consider user perspectives on what constitutes engagement and on how the intervention is best implemented in a clinical setting (MacDonald et al., 2013).

Individually tailored approaches and person‐centered services, prioritizing patient participation in goal‐setting and decision‐making, are widely endorsed (Kjellstrom et al., 2007; Pollock et al., 2014; Yun & Choi, 2019). However, difficulties with implementation are often reported (Busetto et al., 2020; Lloyd et al., 2018), and thus more user‐based knowledge regarding individualization, goal‐setting, and decision‐making in post‐stroke physiotherapy is needed.

Previous research has highlighted expectations of functional improvement and increased levels of activity as reasons why patients appreciate physiotherapy (Pound et al., 1994). The physiotherapist is often viewed as someone who provides knowledge, whose attitude is essential for motivation (Jansson & Carlsson, 2021; Kelly et al., 2020), and a source of faith and hope (Pound et al., 1994). Interestingly, Peiris et al. (2012) found that patients value the interaction with the physiotherapist more than the content of the sessions.

Interaction with others creates meaning (Fuchs & De Jaegher, 2009), and such meaningful engagement can significantly shape the outcome of stroke rehabilitation (Galvin et al., 2009; Levin & Demers, 2020). Indeed, behavioral neuroscience and contemporary models of motor learning suggest that meaningful activities targeting user goals are essential in recovering function (Danzl et al., 2012; Levin & Demers, 2020; Newell & Verhoeven, 2017). The interaction between patient and physiotherapist is, by nature, inherently embodied (Roenn‐Smidt et al., 2020), meaning that the body is conceived as experiencing and expressive simultaneously as being a biological organism (Merleau‐Ponty, 2008). In physiotherapy, interaction and clinical skills are interwoven and embodied, evolving through words, gestures, and hands‐on interactions (Normann, 2020). Given the embodied and interactive nature of physiotherapy, any investigative model must attend to the interaction between physiotherapist and patient, as well as to their motivations, bodily states, capacities, skills, and needs.

Enactive theory in cognitive science draws on phenomenology and dynamic systems theory, viewing the interactions between mind, body, and the environment as inseparably intertwined in mental processes (Thompson, 2007). We propose that this theory is suited to illuminate significant aspects of the interaction between patient and physiotherapist, as it encompasses the way that a person's bodily needs, motivations, and constraints determine how they make sense of their interactions with the world (Thompson, 2007). Two of the most relevant technical concepts here are agency and participatory sense‐making. Agency is defined as a person's adaptive capacity to regulate their interactions with the environment according to self‐generated norms. Cognition, or sense‐making, is defined as a person's participation in what matters to them (Fuchs & De Jaegher, 2009). Socially, people engage in participatory sense‐making; if making sense of the world is deeply determined by how one moves around in it, then engaging with others—including when moving together—means that sense‐making activities are partly co‐determined. Thus, how people understand the world, themselves, and each other—including, what it means to have suffered a stroke and to engage in physiotherapy for recovery—is determined through embodied participation.

The aim of this study was to identify user experiences of I‐CoreDIST and of usual care in post‐stroke physiotherapy by addressing the following research question: How do individuals with stroke experience the bodily and interactive course of physiotherapy during their recovery?

## 2 METHODS

### 2.1 Design

Based on the research question, a qualitative interview within a phenomenological hermeneutic framework was chosen, as it allows knowledge to be derived from lived experiences (Cresswell & Poth, 2018; Malterud, 2015).

### 2.2 Context of the study

**FIGURE 1:**
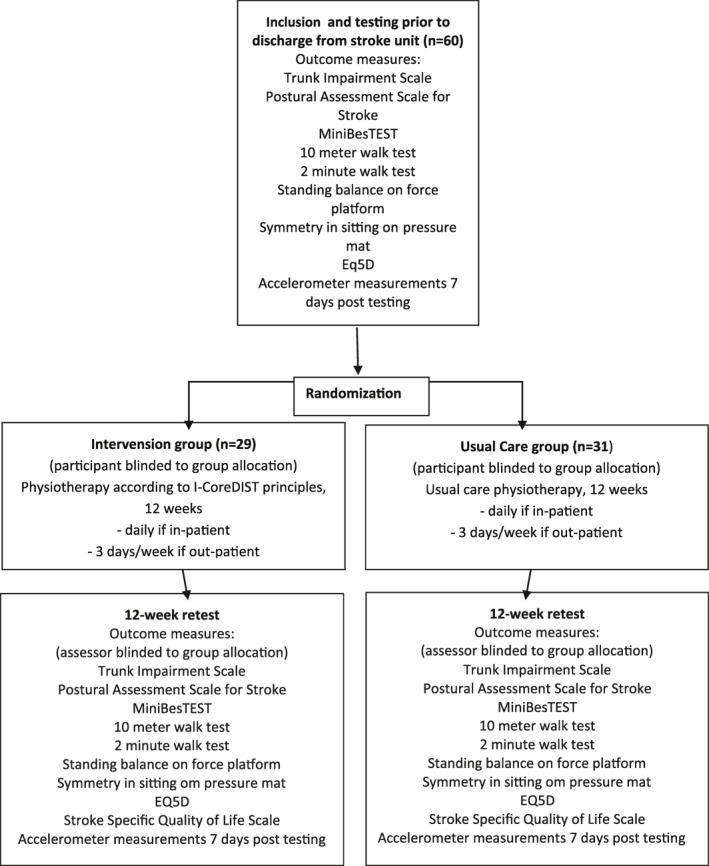
Flowchart of the randomized controlled trial

This interview study was nested within a randomized controlled trial (RCT; Figure 1; ClinicalTrials.gov Identifier: NCT04069767), comparing a new intervention, I‐CoreDIST, against usual care (Table 1). Data were collected between December 2019 and December 2020. Inclusion and exclusion criteria are outlined in Table 2. The first (MS), third (ECA), and last (BN) authors have developed the I‐CoreDIST intervention. They are, together with the fourth author (KBA) investigators in the RCT but have not been involved in the treatment of any participants.

**TABLE 1:** Intervention (I‐CoreDIST) and usual care

Intervention	Usual care
Physiotherapy daily if in‐patient or 3 days/week if outpatient.	Physiotherapy daily if in‐patient or 3 days/week if outpatient.
12‐week follow‐up	12‐week follow‐up
Structure for assessment	No guidelines regarding physiotherapy approach
Clinical reasoning charts	
Booklet containing 44 illustrated exercises, each with five levels of difficulty	

**TABLE 2:** Inclusion and exclusion criteria for the RCT

Inclusion criteria	Exclusion criteria
Admitted to the stroke unit with confirmed stroke	Unable to cooperate in physiotherapy
Age: 18–85	Previously known dementia preventing participation in physiotherapy
Premorbid modified ranking scale 0–3	Ongoing substance‐abuse
Able to sit unsupported for 10 s	Other severe disease preventing rehabilitation
Trunk impairment Scale—Norwegian version score <15	

### 2.3 Participants and sample

Recruitment was conducted at two stroke units. We purposively sampled 19 participants (ID1−ID19) from both the intervention group (IG) and the usual care group (UC), aiming to detect perceptions of content and to identify differences and similarities. To ensure a diverse sample, participants were drawn from a variety of geographical locations, and they differ in gender, age, stroke location, and level of disability but with no severe aphasia. The gender composition differed between the two groups with 20% females in the UG and 67% in the IG. Furthermore, half of the patients in the UG had inpatient rehabilitation compared to only one third in the IG. Median age (75 years) and NIHSS at admission (3), were the same in both groups. Participants' characteristics are shown in Table 3.

**TABLE 3:** Overview of participants

ID	Gender	Age	Type of stroke	Location	Side	Premorbid employmentstatus	NIHSS at admission	In‐patient rehabilitation	Post‐stroke week at interview	Group
1	Male	55	Infarct	Parietal	Right	Employed	0	No	10	C
2	Male	75	Infarct	Frontal	Left	Retired	2	No	9	C
3	Male	78	Infarct	Frontoparietal	Right	Retired	3	Yes	9	C
4	Female	73	Infarct	Temporoparietal	Bilateral	Retired	11	Yes	18	I
5	Female	77	Infarct	Frontal and occipital	Bilateral	Retired	3	No	13	I
6	Male	58	Infarct	Brainstem	Left	Disability benefit	4	No	12	I
7	Male	75	Infarct	Parietooccipital	Right	Retired	4	Yes	27	C
8	Male	77	Infarct	Parietal	Left	Retired	3	No	25	C
9	Female	79	Infarct	Parietal	Left	Retired	1	No	24	I
10	Female	82	Infarct	Frontal	Right	Retired	NA	No	19	C
11	Male	75	Infarct	Parietal	Right	Retired	2	No	25	I
12	Female	39	Infarct	Temporal	Left	Disability benefit	5	Yes	19	I
13	Male	81	Haemorrhage	Parietooccipital	Right	Retired	14	Yes	24	C
14	Female	71	Infarct	Internal capsule	Right	Retired	3	Yes	15	C
15	Male	62	Infarct	NA	Left	Disability benefit	4	Yes	38	C
16	Male	74	Infarct	Temporal	Left	Retired	2	No	29	C
17	Male	75	Infarct	Corona radiata	Bilateral	Retired	3	No	7	I
18	Female	81	Infarct	Cerebellum	Left	Retired	0	No	22	I
19	Female	81	Infarct	Internal capsule	Left	Retired	4	Yes	9	I

Abbreviations: C, control group; I, intervention group; NA, not available; NIHSS, National Institute of Health Stroke Scale.

We initially aimed at interviewing participants 6–12 weeks after inclusion but encountered considerable delays in recruitment caused by the COVID‐19 pandemic. Due to reduced capacity for testing at the hospitals and a ban on out‐patient physiotherapy treatment, inclusion was stopped between March and June 2020. To maintain progress in the project, participants were sampled from the initial 40 participants in the RCT, rather than from the full sample (recruited between September 2019 and September 2021). As a result, some were interviewed up to 38 weeks after inclusion. When analysis did not reveal new themes, we concluded that saturation was reached, and that the data possessed adequate information power (Malterud et al., 2016).

### 2.4 Data collection

The interviews were conducted by MS and lasted between 20 and 91 min, constituting a total time of 840 min. The first six interviews were face‐to‐face, while the rest were, due to COVID‐19 restrictions, performed over the phone, using a speakerphone and a digital recorder. A theme‐based interview guide with open‐ended questions initiated reflections on: (1) the content and experience of physiotherapy, (2) the participation in decision‐making and goal‐setting, and (3) the interaction/relationship with the physiotherapist. Communicative validation was ensured by asking follow‐up questions, rephrasing, and requesting details of positive and negative experiences (Brinkmann & Kvale, 2015). A debrief revealed no negative experiences.

### 2.5 Data analysis

All interviews were transcribed verbatim by MS and a secretary not otherwise connected to the project. Data were coded using NVivo software, v12.6.0 (QSR International, 2019) and analyzed through systematic text condensation, a process of decontextualization and recontextualization (Malterud, 2012). The analysis followed four steps: (1) MS read every interview, while ECA and BN reviewed a selection to develop an overall impression. This process was followed by discussions on preliminary themes, (2) MS identified meaning units containing information about the research question and organized these into code groups, (3) Each code group was sorted into subgroups, and the contents were reduced into a condensate written in first person and illustrated with quotes. Condensates were read by MS, ECA, and BN. Interpretations, informed by the theoretical framework, were discussed with the second author (HDJ), and (4) Each condensate was rewritten as an analytical text in third person and then validated to ensure that the syntheses of the data reflected the original context. All authors reviewed, revised, and discussed the final manuscript. An example of the analysis process is depicted in Table 4. The analysis generated three categories, each with two subgroups (Table 5).

**TABLE 4:** Examples of the analysis process

Step 1, preliminary themes	Step 2, examples of meaning units	Step 3, code group, *sub‐groups, and condensates (excerpts)*			Step 4, category and analytical text
Trust To be challenged Support Receiving feedback Positivity	*It is important that they take this seriously, that they find it important* *I trusted her a lot, she was good at making me do the exercises, even when I thought I couldn't* *That they show engagement, that's the most important thing. They look after you, you know* *He made these tiny changes and suddenly I could do the same exercise without pain*	Relations and roles	*Trust in professional knowledge*It felt important that he looked after me, that he suggested adjustments when some exercises hurt a bit. It helped straight away, I did the same exercise with no pain. You have to believe in what they are doing, that they are doing the right thing to help you get better and you need to do as you are told. I guess they knew what was right to do, that they have seen me. She came up with lots of things I would never have thought of. She was incredibly skilled at spotting my weaker points	*Engagement, presence, and feedback*	Interaction: Supportive and demanding
				The comments from the physiotherapist saying “you did this better than last week” really created the motivation to continue. That she made me feel a certain progress throughout this period, and that she seemed to care. That you're not just there as a thing, but as a person. I Get motivation from being pushed and from their guidance. It means a lot. If he hadn't been there and payed attention I wouldn't have worked so hard. That they support med and give positive feedback. I Was a bit depressed from time to time and the physiotherapist was particularly good at motivating me	Participants view trust as the most important aspect of their interaction with the physiotherapist. Trust was mainly brought forward in the context of having trust in the physiotherapist's professional opinions and decisions made regarding their treatment. It was also important to feel able to trust that the therapist was honest. Participants valued their physiotherapist professional opinion and wanted to be challenged, pushed and corrected in therapy. Simultaneously they found it important that the physiotherapist was supportive, understanding and someone they could talk to

**TABLE 5:** Categories and subgroups

Categories	Subgroups
Explicit or embedded: Diversity of approaches	Assessment, a tool for the physiotherapist
	Goal‐setting, tacit or spoken
Interventions and perceived bodily changes: Function and fitness	General and individualized
	Meaningful exhaustion
Interaction: Supportive and demanding	Trust and professional knowledge
	Engagement, presence, and feedback

### 2.6 Research team and reflexivity

In aiming for transparency, we have adhered to the Standards for Reporting Qualitative Research (SRQR; O'Brien et al., 2014). Reflexivity was maintained through the preparation, analysis, and writing by discussing and challenging our established assumptions. BN, ECA, and MS are physiotherapists, KBA is a neurologist, and HDJ is a philosopher with expertise in enactive theory. The physiotherapy and neurology background provided the group with varied positioned insights (Paulgaard, 1997) that assisted MS, ECA, BN and KBA with creating the interview guide—a process in which a user representative participated. The group's positioned insights, along with HDJ's outsider perspective, facilitated multiple interpretations. None of the authors were personally or professionally acquainted with any of the participants.

### 2.7 Ethical considerations

The study was approved by the Regional Committee for Medical Research Ethics North Norway (REK North: 2017/1961). Informed, written consent for participation in the RCT and for the interview study (if selected) was obtained from all participants, and data were anonymized. Consent was verbally confirmed prior to interviews.

## 3 RESULTS

### 3.1 Explicit or embedded: Diversity of approaches

#### 3.1.1 Assessment: A tool for the physiotherapist

Descriptions of the initial encounter with the physiotherapist ranged from having a conversation, testing of strength and balance, to no formal assessment. Some assessments were thorough while others were perceived as superficial or partial. Two participants, for example, reported an assessment of only the affected body part, such as a paretic hand. Those participants who did not report a formal assessment, still perceived one integrated into their treatment. They construed the physiotherapist's observations as the basis for the assessment:

*We talked a little and then I think we did a couple of exercises. I suppose she needed an introduction to figure out what I was able to do and where I stood* (ID17, 75 years old, IG).

Regardless of approach, the participants in both groups trusted the physiotherapists' professional choices and expressed little approval or disapproval. They described these encounters in neutral terms and seemed to acknowledge the initial assessment as being for the physiotherapist, rather than for themselves.

#### 3.1.2 Goal‐setting: Tacit or spoken

Goal‐setting also varied within groups. In the rehabilitation units, this process often occurred in a multidisciplinary context where the patients actively voiced their thoughts about goals and priorities. This was viewed positively, one participant describing it as being heard and the focus of attention. Such explicit processes were not recounted amongst those who had physiotherapy in the municipalities. Sixteen participants reported having reflected upon their personal goals, such as “getting better” or returning to their previous level of function. Interestingly, among the 11 participants who had not received in‐patient rehabilitation, 8 had never spoken about goals with their physiotherapist.

*I had no idea if there were any goals, I guess it was to make me improve my function, she (the physiotherapist) decided on what to do, I would not know why to choose which exercise. I was just happy to get the physiotherapy* (ID18, 81 years old, IG).

When interventions were targeted and tailored to the users' needs, this was interpreted as a tacit, mutual understanding with regards to the aim of therapy. Only one respondent found that the lack of explicit goals reduced their motivation for physiotherapy.

### 3.2 Interventions and perceived bodily changes: Function and fitness

#### 3.2.1 General and individualized

Participants in both groups reported that the physiotherapist chose the content of the sessions, and that balance‐, gait‐, and stair‐training were central elements. Group differences were more distinct in the accounts of content and mode of delivery. The participants in the IG described mainly one‐on‐one therapy utilizing bodyweight exercises, sensory stimulation supported by hands‐on interactions, and verbal explanations as outlined in the intervention guidelines. Several perceived immediate changes during a therapy session, which they found surprising. They frequently demonstrated knowledge about the purpose of exercises (i.e., that the intervention targeted core strength) and largely spoke of improvements in terms of felt bodily changes.

*We talked about the exercises and which muscles we used. I felt more in contact with my body, that I used the muscles around my pelvis and back. They make me stronger, my balance is better and I have more control over my arm and leg* (ID19, 81 years old, IG).

In the UG, measures were also mostly exercise‐based, yet more often performed in a gym utilizing a mix of bodyweight exercises, apparatuses, weights, and endurance‐training equipment. Approaches were structured around interval‐based training or repetitions and sets. Progress was generally measured through increased resistance or number of repetitions.

*He said I was weaker in one leg and that we were going to make it as strong as the other. I was to use the leg press‐machine. I started doing 45 kg, then 60 kg and now I am doing 65 kg* (ID10, 82 years old, UG).

Positive bodily changes were reported by 15 participants across groups, and were the most important factor in maintaining motivation. Individual tailoring, variations, and gradual progressions in tasks and exercises were appreciated and interpreted as evidence of progress toward their goals.

*I am in much better shape now than I was before the stroke. It must be the training, I'm sure. I am stronger and it is easier to walk, I hardly use my walker anymore* (ID5, 77 years, IG).

Progress in this context comprised not only regaining bodily control or functioning in ADL, but also gradual improvements in general strength and endurance. One participant felt that the exercises did not suit them since they differed greatly from their previous experience of passive treatments in physiotherapy.

### 3.3 Meaningful exhaustion

The majority of participants wanted to be challenged, pushed, and corrected in therapy to bring about progress and a feeling of achievement. High‐intensity training generated optimism, as the exertion was interpreted as a sign of normality, or that “the body is working.”

*He always tried to get some momentum into what we were doing. He tried to get across that if you don't push yourself, if you don't try then nothing will happen. He didn't say it but it was there in the way things were done* (ID15, 62 years old, UG).

Repetitive training and exercises that were insufficiently targeted or challenging were depicted as negative features that diminished their commitment.

*I got bored with it. It was always the same, we did the same tasks every time. It made it easier for me to say bye* (ID12, 39 years old, IG).

Eleven participants reported feeling very tired for one or two hours after physiotherapy, particularly in the early stages. For most, this eased as their endurance improved, which made some feel more positively about physiotherapy.

*No matter how fatigued I felt that day, once I got to the physiotherapy clinic I just did it. I would not have been able to cope with the music or noise in a normal gym. My physiotherapist made me work really hard for a whole hour and I felt fine* (ID10, 82 years old, UC).

The tiredness following physiotherapy is differentiated from the daily fatigue with which several of the participants struggled. One said that, due to their history, they had not thought it possible to experience such progress, and they were now keen to see how far training could take them.

### 3.4 Interaction: Supportive and demanding

#### 3.4.1 Trust in professional knowledge

Trust was viewed as the most important aspect of the interaction with the physiotherapist. Two main features were highlighted: (1) trust in the physiotherapist's professional opinions and decisions and (2) feeling safe to be personal in the interaction. All participants trusted the physiotherapist's knowledge and abilities.

*If they had not shown engagement like they did I wouldn't have known what to do. That would have been my biggest problem. I wouldn't have known how to get out of that wheelchair. They worked gradually, every step seemed unachievable initially, and then you manage. I could not have done that alone* (ID14, 71 years old, UG).

Two participants were told by their physiotherapist that their goals were unrealistic. Both initially felt disappointed, but they ultimately appreciated the honesty and respected the professional evaluation.

### 3.5 Engagement, presence, and feedback

The participants valued that their physiotherapist showed commitment, exhibited a supportive and understanding attitude, and served as someone they could talk to. One described his physiotherapist as “fun and serious” and found both features important, along with the physiotherapist being “a bit psychologist.” The role of the physiotherapist as an engaging motivator who expresses engagement during challenging times was recognized as essential, and their feedback during sessions was emphasized as crucial.

*You wouldn't put your soul into it like you do when you hear: Awesome! good job! or things like that. Then you know that you are doing your best* (ID2, 75 years old, UG).

The participants valued the physiotherapist's feedback, whether in the form of verbal praise or through verbal and/or tactile cues provided during specific movements or exercises.

*She held me and pushed me forward at the same time in a way that made me straighten up my upper body. It is like it did something to me, immediately after she finished. It felt like I could walk better* (ID14, 71 years old, UG).

The physiotherapist's presence was deemed important, even when simply checking in on participants at the gym. Many feared that they would not be able to maintain their achievements independently.

## 4 DISCUSSION

The participants in this study revealed that interaction with the physiotherapist, which was guided by felt bodily changes, ranged from formal/explicit to tacit/implicit. Experiencing positive bodily changes, along with tailored difficulty and intensity in training, were essential contributors to the development of meaning and motivation, regardless of approach. In the interaction between the patient and the physiotherapist, the latter set the parameters for what to do and how to do it. Trust and engagement were also paramount and multifaceted in this context, involving both interpersonal skills and professional expertise.

### 4.1 Embodiment: The missing link

An embodied approach appeared to be more integrated into goal‐setting and treatment than into assessment. Participants commonly saw the assessment as being evaluated, rather than playing an active role. It seems that when the body is viewed from a third‐person perspective as a biological and biomechanical system, rather than as an embodied self, an opportunity is missed for an interactive approach to assessment—one that could edify both patient and therapist and could clarify how underlying impairments influence movement problems (Normann, 2020). Previous research has shown engagement and sense‐making to be facilitated through felt bodily changes (Normann et al., 2013). Our results highlight the need to make the assessment not simply a baseline for the physiotherapist's clinical reasoning, but also a relevant and meaningful learning opportunity for the patient.

In the literature, goal‐setting processes are often treated as single activities isolated from other rehabilitation processes (Plant & Tyson, 2018), with many barriers to their implementation (Lloyd et al., 2014). In our material there was a marked difference between how goal‐setting was carried out in multidisciplinary in‐patient settings and monodisciplinary out‐patient settings. In contrast to the current literature advocating SMART^2^ goals for such processes (Plant & Tyson, 2018), our results suggest that goal‐setting, particularly in the one‐on‐one setting in practice, is often tacit and implicit—and to a larger extent evaluated/confirmed through felt bodily changes. In the multidisciplinary team setting, explicit goals seemed to have a more overarching, coordinative function. Yet they are still confirmed and evaluated by bodily changes in the day‐to‐day therapist–patient interaction. Our findings are supported by research suggesting that goal‐setting is not separate from the treatment situation, but rather interlinked and integrated—and thus often under‐documented (Jung et al., 2017). For the written or verbally set goals to make sense, there is a need for coherence between these and the embodied experiences of the therapy situation.

There were differences between groups in their descriptions of content, their understandings of the purpose behind the exercises, and in their accounts of what constituted progress. Felt bodily change is a key to engagement and sense‐making, although expressed in different forms. While the participants in the IG spoke of the progress they made in terms of regaining control of their bodies, those in the UG measured progress more in terms of external, quantitative measures. Regardless of approach, it is vital that progress—the gradual increase in difficulty vis‐à‐vis the patient's goals—even if not explicit, makes sense, creates engagement, and facilitates meaning‐making processes. It seems that there was a stronger emphasis on specificity and on awareness of purpose in the IG. However, focus on strength and endurance training, such as that expressed in the UG, is recommended (Saunders et al., 2020). A combination of approaches, providing both specificity and intensity, as endorsed by Pollock et al. (2014), should also be feasible. The improvements in strength and endurance were particularly significant during plateaus in recovery of activity of daily living (ADL). Such improvements served as a confirmative link between effort and gains, and as such made the endeavor and exhaustion meaningful. It is noteworthy that the exhaustion following exercise was well tolerated and essentially perceived differently than that associated with fatigue, which more often was related to noisy environments or social settings with which several participants struggled in their daily lives. Our findings point to how embodiment and the co‐construction of meaning, occurring through verbal and nonverbal actions and physical interactions, are integrated in physiotherapy practice.

### 4.2 Interaction

Interactions, such as that between a patient and a physiotherapist, are always shaped by self‐regulated norms and established power‐relations. The participants make sense of each other, their actions, and their surroundings together through participatory sense‐making (Fuchs & De Jaegher, 2009). Trust is central to this process. The expectations of how an interaction with a physiotherapist would proceed are, in enactive terms, part of a participation genre (Di Paolo et al., 2018). The patient's acceptance of the physiotherapist as the decision‐maker in this interaction, is also part of such a genre. The fact that a physiotherapist possesses the adequate professional knowledge and will make optimal decisions on the treatment is implicitly assumed and functions as a premise for the interaction (Roenn‐Smidt et al., 2020). The physiotherapist's role is complex, as they are also expected to provide emotional support, as well as to motivate and to push the patient with regards to intensity in training. When fulfilling these expectations, the physiotherapist is the regulator of the interaction. We found that the patient's expectations for physiotherapy are mainly connected to the physiotherapist's traits and not to the specific content of therapy. Our results are in line with those by Sheppard et al. (2010), who found that such traits are often referred to as the physiotherapist's personal characteristics, yet in practice are impossible to distinguish from their professional manner, since motivation and communication skills are part of the professional role. The patient's own role in the interaction is to exert the required effort. Although patients seemingly “do as they're told” in physiotherapy, they are autonomous participants and active agents; they possess the capacity to regulate the interaction through their efforts. If the central criterion of experiencing positive bodily changes is not met, the patient may become the regulator of the encounter by reducing their engagement and efforts or by withdrawing from therapy entirely.

### 4.3 Limitations

This study was conducted in two regions in Norway, limiting the findings to the Scandinavian health care system. The main features of physiotherapy treatment, however, are shared internationally, and applying concepts from enactive theory serve as a theoretical generalization (Malterud, 2015). We sampled participants strategically, aiming for a broad sample, but cannot rule out the possibility that excluded participants may have been able to add valuable contributions. Furthermore, the criteria for participation in the RCT influenced the sample, as these excluded those with more severe disabilities. Some interviews were delayed, which might have interfered with the participant's ability to remember events and perhaps introduced recall bias. Our impression, however, was that most participants recalled the events clearly**.**

## 5 CONCLUSION

This study highlights how embodiment, along with the co‐construction of meaning that occurs through verbal and nonverbal actions and physical interactions, are integral to physiotherapy practice. Experiencing bodily changes and exertion from post‐stroke training can facilitate sense‐making, galvanize commitment, and inspire a positive attitude toward physiotherapy. Trust is an essential part of the interaction between patient and physiotherapist. Patients find that a physiotherapist's ability to apply professional knowledge, to motivate their patients, and to provide emotional support are fundamental aspects of their role.

### 5.1 Implications for physiotherapy practice

‐ Assessments must be made meaningful and instructive for patients—a process that is facilitated by recognizing the body as the locus of experience and expression. Simultaneously as being a biological organism.
‐ Improvements in general fitness contribute significantly to maintaining motivation during plateaus in ADL‐recovery.

## CONFLICT OF INTEREST

The authors declare no conflict of interest.

## AUTHOR CONTRIBUTIONS

Britt Normann, Marianne Sivertsen, Karl Bjørnar Alstadhaug, and Ellen Christin Arntzen contributed to the design of the study. Marianne Sivertsen conducted the interviews. Marianne Sivertsen, Britt Normann, and Ellen Christin Arntzen contributed to the analysis of the data. Marianne Sivertsen drafted the manuscript. All authors contributed to the interpretation of data, critically revised the manuscript and gave final approval.

## Data Availability

Research data are not shared.

## ENDNOTES

^1^
I‐CoreDIST: Individualized Core activation combined with DISTal functional movement. I = individualized, Core = trunk, D = dual task, I = intensive, S = specific, stability, somatosensory stimulation, and T = teaching, training.

^2^
SMART = Specific, Measurable, Achievable, Realistic, Timely.
